# Histone variant H3.5 in testicular cell differentiation and its interactions with histone chaperones

**DOI:** 10.1038/s41598-024-83206-9

**Published:** 2024-12-19

**Authors:** Patrick Philipp Weil, Anton Pembaur, Beatrice Wirth, Eda Oetjen, Hannes Büsscher, Klemens Zirngibl, Malte Czarnetzki, Stella Braun, Jann-Frederik Cremers, Daniel Gödde, Stephan Degener, Jan Postberg

**Affiliations:** 1 Clinical Molecular Genetics and Epigenetics, Faculty of Health, Centre for Biomedical Education & Research (ZBAF), Witten/Herdecke University, Alfred-Herrhausen-Str. 50, 58448 Witten, Germany; 2 Centre of Reproductive Medicine and Andrology, University Hospital of Münster, Münster, Germany; 3 Chair of Pathology, Centre for Clinical and Translational Research (ZFKM), Helios University Hospital Wuppertal, Witten/Herdecke University, Heusnerstr. 40, 42283 Wuppertal, Germany; 4 Chair of Urology, Centre for Clinical and Translational Research (ZFKM), Helios University Hospital Wuppertal, Witten/Herdecke University, Heusnerstr. 40, 42283 Wuppertal, Germany

**Keywords:** Spermatogenesis, Histone variants, Epigenetic regulation, Histone chaperones, Chromatin remodeling, Epigenetic Plasticity, Cell biology, Epigenetics

## Abstract

Testicular cell differentiation is a highly regulated process, essential for male reproductive health. The histone variant H3.5 is apparently a critical player in this intricate orchestra of cell types, but its regulation and function remains poorly understood. To elucidate its role, we fractionized testicular cells using c-Kit/CD117 as a separation marker and analyzed H3.5 expression. Further, we investigated the regulation of H3.5 expression using public data repositories. We explored DNA methylation patterns in specific regions of the H3-5 gene and assessed H3-5 copy number gain in seminoma specimens. Additionally, we examined the testicular localization of H3.5 and its histone chaperone interactions to understand its regulation at the protein level. We used qRT-PCR, MeDIP, and qPCR to study H3.5 expression and DNA methylation in various cell types. H3-5 copy number gain was analyzed using qPCR. Protein interactions were investigated through fluorescence-2-hybrid assays in baby hamster kidney cells. H3.5 is primarily enriched in spermatocytes. DNA methylation of a CpG island overlapping the H3-5 promoter appeared to be involved in the tissue-specific regulation of H3.5 expression. Elevated H3.5 expression was observed in seminoma specimens, suggesting a potential link to testicular tumors. H3-5 copy number gain was associated with elevated H3.5 expression in seminoma specimens. Furthermore, we identified physical interactions between H3.5 and histone chaperones Asf1a and Asf1b, HIRA, CAF p150 and DAXX, shedding light on the protein-level regulation of H3.5. These findings provide valuable insights into the molecular mechanisms governing testicular cell differentiation and the potential role of H3.5 in testicular pathologies.

**Supplementary Information:**

The online version contains supplementary material available at 10.1038/s41598-024-83206-9.

## Introduction

**Fig. 1:**
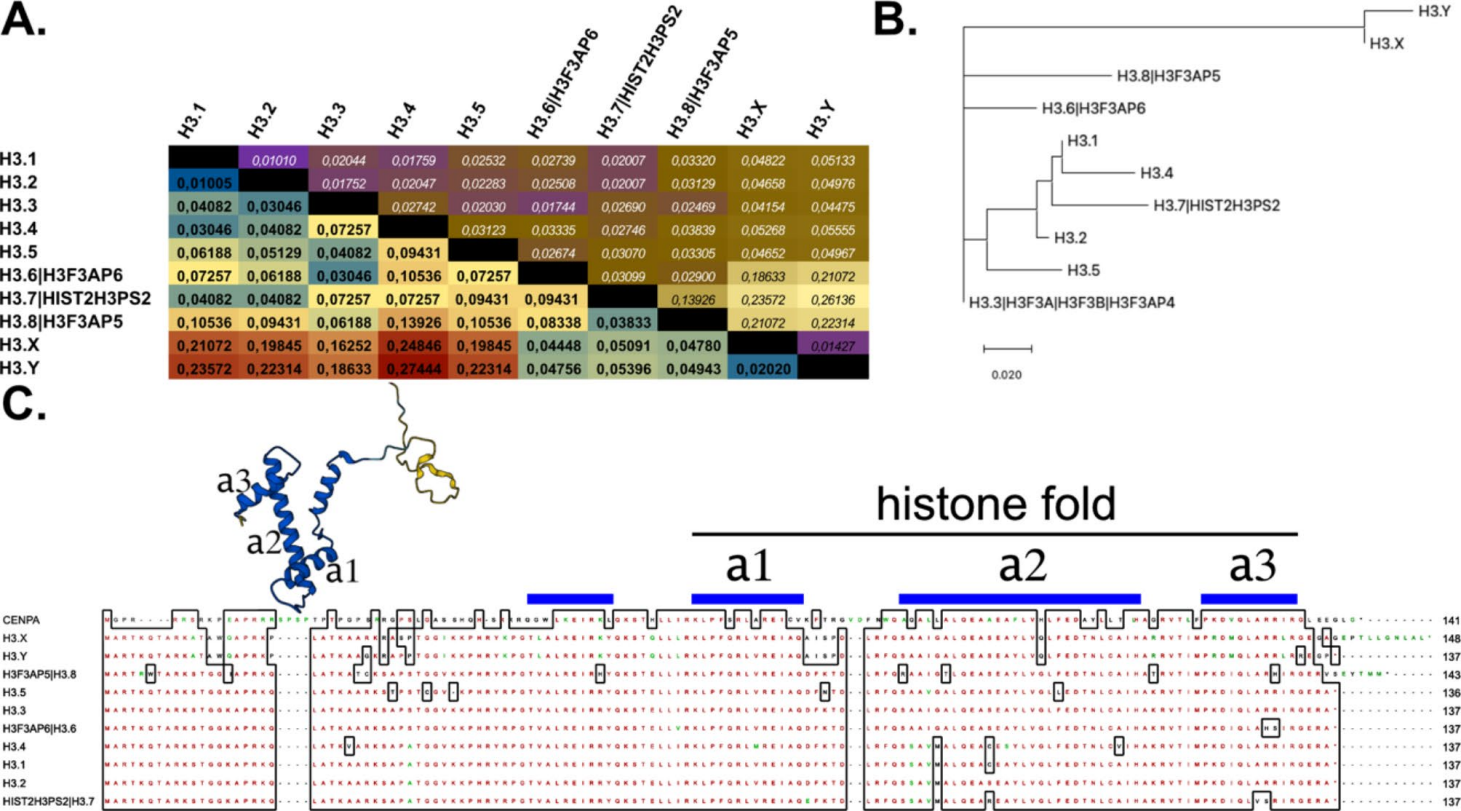
Evolutionary distance analyses of human H3 variants. (**A**) The number of amino acid substitutions per site from between sequences are shown. Standard error estimate(s) are shown above the diagonal. Analyses were conducted using the Poisson correction model^8^. The analysis involved 16 amino acid sequences. All ambiguous positions were removed for each sequence pair. There were a total of 105 positions in the final dataset. Evolutionary analyses were conducted in MEGA7^9^. (**B**) The evolutionary history was inferred by using the Maximum Likelihood method and JTT matrix-based model^10^. The tree with the highest log likelihood (-530.27) is shown. Initial tree(s) for the heuristic search were obtained automatically by applying Neighbor-Join and BioNJ algorithms to a matrix of pairwise distances estimated using the JTT model, and then selecting the topology with superior log likelihood value. This analysis involved 10 amino acid sequences. There were a total of 100 positions in the final dataset. Evolutionary analyses were conducted in MEGA X^11,12^. (**C**) Alignment of 11 histone H3 variants.

The male reproductive system is a marvel of cellular differentiation, orchestrated with precision to ensure the production of spermatozoa, the vehicles of genetic information transfer from one generation to the next. In this intricate process, the testis plays a central role, housing an array of cell types that collaborate in the generation and maturation of sperm cells. One of the key processes during spermatogenesis is the dramatic reorganization of chromatin in progenitor cells, resulting in the replacement of most – not all - nucleosomes by protamines in mature sperm cells, and eventually leading to the ultra-compact nuclei of sperm^1^. Here, similar to other developmental processes, histone variants play critical roles in chromatin structure and gene regulation^2–4^. In many taxa variants of H2A, H2B, and H3 exist, although histones are among the most conserved eukaryotic proteins. Most of these histone variants differ by only a few substitutions or indels of individual amino acids. The human histone H3 variants H3.1, H3.2, H3.4, and H3.5 differ by only 4–8 positions from the 136 aa variant H3.3, which is evolutionarily likely ancestral^5^. Many differences between individual variants are observed in positions in the unstructured N-terminus. Here, some specific amino acids are either substituted or deleted, which can serve as direct targets for covalent post-translational modifications (PTMs). Alternatively, subtle differences in recognition motifs of PTM ‘readers’ can lead to their differential binding, thus influencing chromatin structures^6^. Besides the unstructured N-terminus, variant-specific variations within the structured histone fold can contribute to specific interactions of H3 variants with different histone chaperones^7^ (Fig. 1).

Taken together, there is a growing consensus that nearly every single amino acid in histones is functionally relevant – “every amino acid matters”^13^, and the balanced regulation of histone variants and histone chaperones, as well as deviations due to mutations, can be extremely important, especially in developmental processes and the development of tumor diseases^14,15^.

Among the recognized key players in the testicular cellular ensemble are histone H3.4 and H3.5, variants of the core histone protein H3, as well as testis-specific H2B (TSH2B)^16,17^. Interestingly, a H3.4 homolog (H3t) in mice spermatogenesis is involved in the processes, which enable male germ cells to enter meiosis^18^. In the context of the testis, hominoid-specific histone H3.5’s involvement in the differentiation of testicular cells has also been coming into focus^19,20^.

To shed further light on the role of H3.5 in testicular differentiation, this study embarks on a multifaceted exploration. We utilize c-Kit/CD117 as a marker to fractionize testicular cells and examine the distribution of H3.5 expression. Further investigations involve the development of specific antibodies for H3.5, leading to an in-depth understanding of its nuclear localization in different cell types. We also assessed DNA methylation patterns around the H3-5 gene. Moreover, the potential influence of H3-5 copy number gain in the context of elevated H3.5 expression in testicular tumors is examined. By unraveling the multifaceted regulatory mechanisms of H3.5, this study contributes to the broader understanding of testicular cell differentiation and provides insights into its potential roles in the context of testicular pathologies. The journey begins with an exploration of the intriguing roles played by H3.5 in the testis, offering a glimpse into the complexity of male reproduction.

## Results

### Unveiling H3.5 distribution in testicular cells

Our study commenced by fractionating testicular cells using C-Kit protooncogene (c-Kit)/CD117 as a separation marker, since in testis c-Kit protein is reported being expressed in pachytene spermatocytes. This tyrosine kinase is significant in the development and maturation of spermatogonial stem cells during different stages of spermatogenesis and gametogenesis. Its expression is essential for the maturation of primordial germ cells and is dynamically regulated during postnatal spermatogenesis, being present in early-stage cells but absent in later cells of sperm maturation^21^. According to another source, however, a peak of expression rather appears to be associated with the spermatogonial stem cells^22,23^.

**Fig. 2:**
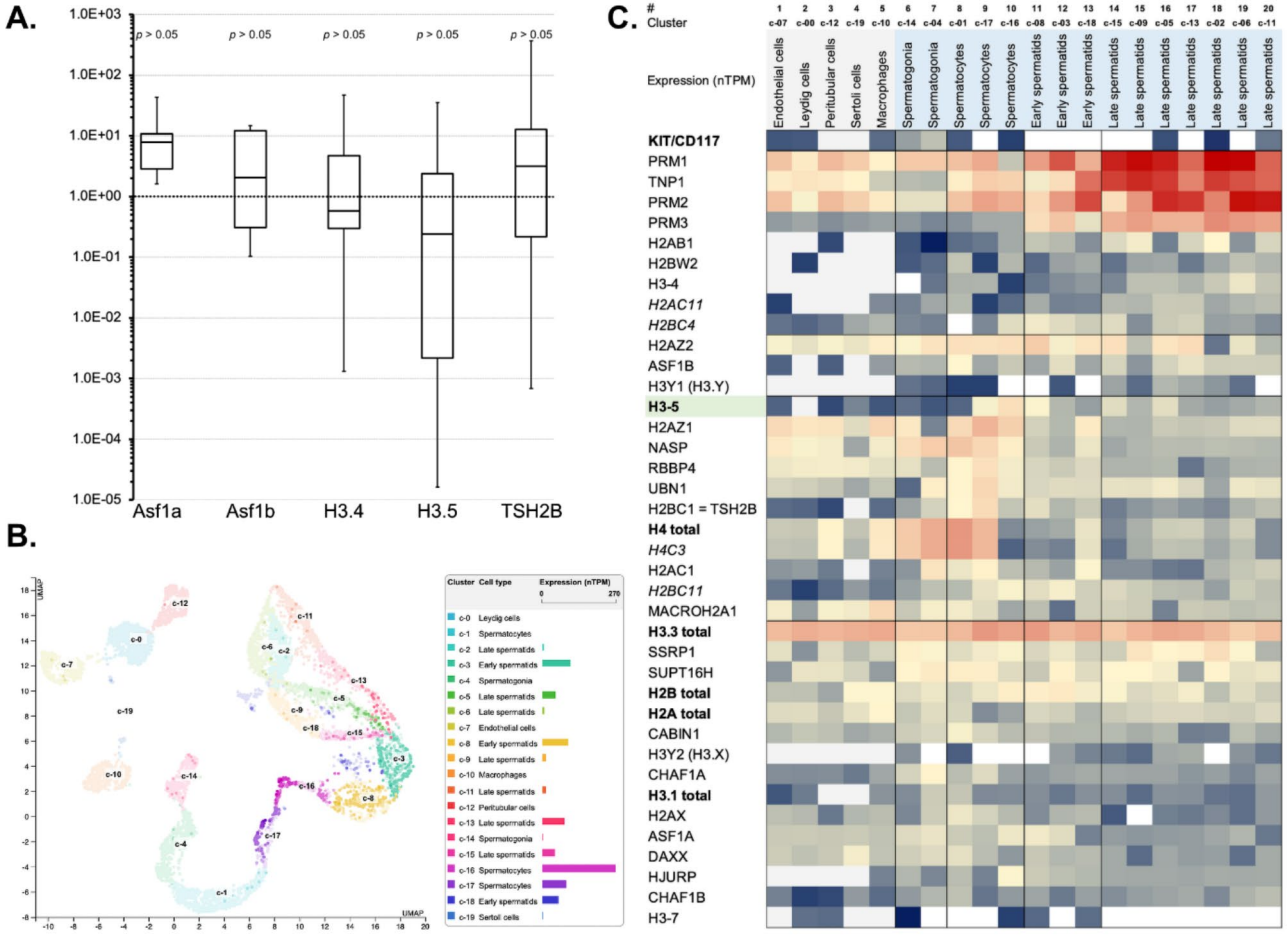
(**A**) Box plot diagram of the expression analysis after cell type separation by MACS technology. Y-axis: Fold change of mRNA expression in c-Kit/CD117-negative vs. positive cells, whereby fold changes > 1.0E + 00 mean relative upregulation in c-Kit/CD117-negative cells and fold changes < 1.0E + 00 mean relative upregulation in c-Kit/CD117-positive cells. (**B**) H3-5 expression (scRNAseq-data) associated with 20 testicular cell type-clusters (c-0 – c-19). (**C**) The heat map illustrates the enrichment of H3.5 mRNA (scRNAseq-data) and mRNAs from other available histone variant genes, multiple histone chaperones, protamines, and other genes of interest associated with 20 testicular cell type-clusters. With respect to spermatogenesis, non-germline cells were separated on the left side of the heat map, and spermatogenesis stages were serially ordered from left to right. Shades of red represent high expression levels, shades of yellow represent moderate levels, and shades of blue represent low expression levels. White indicates the absence of expression data.

From both the separated c-Kit/CD117-positive and the negative cell fractions we purified whole RNA and subsequently performed quantitative reverse transcription PCR (qRT PCR). This approach revealed H3.5 mRNA being somewhat enriched in the c-Kit/CD117-positive fraction with the respect to the c-Kit/CD117-negative fraction, which should contain Leydig cells, Sertoli cells as well as later spermatocyte and spermatid stages (Fig. 2A). This differential expression of the H3.5 mRNA suggests a functional role of this Histone H3 variant in earlier spermatocyte development, i.e. in spermatogonia or/and early spermatocytes.

To gain more detailed insights, we utilized an available data resource comprising single-cell RNA sequencing (scRNAseq) data from 31 major healthy tissues and organs, comprising 557 individual cell type clusters. This scRNA-seq dataset was retrieved from published studies and single-cell databases, including the Single Cell Expression Atlas (https://www.ebi.ac.uk/gxa/sc/home), the Human Cell Atlas (https://www.humancellatlas.org), the Gene Expression Omnibus (https://www.ncbi.nlm.nih.gov/geo/), the Tabula Sapiens (https://tabula-sapiens-portal.ds.czbiohub.org/), the Allen Brain Atlas (https://portal.brain-map.org/) and the European Genome-phenome Archive (https://www.ebi.ac.uk/ega/)^24^. From this comprehensive resource, we extracted normalized read counts from 20 testicular cell type-clusters (c-0 – c-19) for all available histone variant genes, multiple histone chaperones, protamines, and other genes of interest (Fig. 2B, C). Notably, we cumulated read counts for H3.1, H3.3, H2A as well as H2B, since multiple human genes encode each of these histone variants.

The highest expression of H3.5 can be clearly observed in spermatocytes, with notable levels also partially visible in early and late spermatids. However, for H3.5 and H3.4 as testis-specific histone H3 variants, it is important to note that while their phased enrichment is evident, they never achieve predominance over H3.3 (Fig. 2C). This is because H3.3 mRNA is expressed much more strongly throughout the entire duration of spermatogenesis compared to these two H3 variants and other histones. Interestingly, some histones and histone chaperones exhibit similar spatial-temporal mRNA expression patterns to H3.5, such as H2AZ1, H2BC1 (TSH2B), as well as NASP, RBBP4, and UBN1. It is also noteworthy that H3.4 is expressed later than H3.5, primarily in the later stages of spermatids. By the early stage of spermatids, the expression of protamines, such as PRM1, PRM2 and PRM3 as well as transition protein 1 (TNP1), dominates.

### Specific antibody development and immunofluorescence insights using human normal testicular sections

To further explore the protein level of H3.5 expression, we pursued the development of specific antibodies through rabbit immunization, successfully obtaining one responsive antibody (Supplemental information 1). This antibody reacted with a ~ 17 kDa protein band in Western analyses using a normal human testis sample. It reacted also with a ~ 17 kDa protein after E. coli expression of 6-His-H3.5 via pRSET B, but, notably, also with several bacterial proteins of higher molecular weight. Importantly, anti-H3.5 did not react with a mixture of histones H1, H2A, H2B, H3 and H4 from calf thymus (Supplementary Information 1: Figure SI1_F, Figure SI1_G). Subsequently, we applied the anti-H3.5 antibodies and for comparison anti-pan-H3 antibodies, that could not distinguish between H3 variants, for histological analyses of human testicular tissue.

In order to investigate the cellular architecture of human testes during spermatogenesis, serial histological sections of normal human testicular tissue were prepared. These sections were specifically oriented to highlight the radial arrangement of the different stages of germ cell maturation within the seminiferous tubules. This radial organization is a characteristic feature of spermatogenesis, allowing for the precise identification of various cell types involved in this process. Experienced histologists, pathologists, and urologists can reliably distinguish key cell types—including Leydig cells, Sertoli cells, spermatogonia, spermatocytes, spermatids, and mature sperm—based on their specific spatial arrangement, distinctive morphology, and nuclear chromatin patterns. This approach enables detailed cellular characterization without the need for additional immunohistochemical staining, as the intrinsic histological features provide sufficient information for accurate identification in normal tissue.

**Fig. 3:**
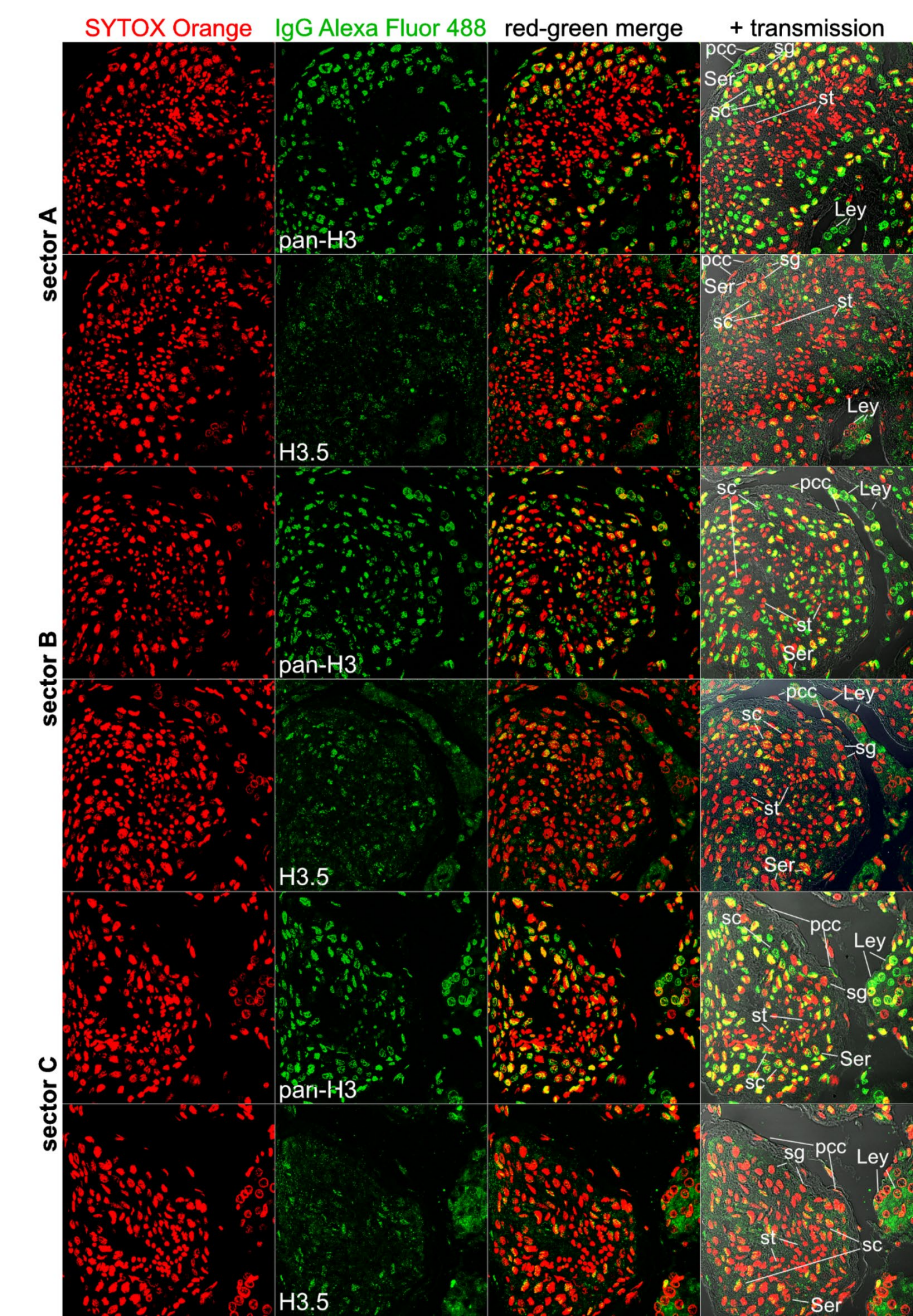
Immunofluorescence microscopy analyses on the localization of pan-H3 or H3.5 in human normal testis sections. In order to compare similar areas (sectors **A**-**C**), we analyzed consecutive microscopic thin sections, each loaded with the anti-H3.5 antibody or a polyclonal anti-pan-H3 antibody, which cannot distinguish between different H3 variants. Red: DNA counterstaining using Sytox Orange dye. Green: H3 staining using goat anti-rabbit pAb conjugated with Alexa Fluor 488. Abbreviations: **pcc**, *peritubular contractile cells/myoid cells*; **Ser**, *Sertoli cells*; **Ley**, *Leydig cells*; **sg**, *spermatogonia*; **sc**, (primary) *spermatocytes*; **st**, *spermatids*.

Applying the anti-H3.5 polyclonal antibody for immunofluorescence microscopy on human normal testis sections, our analyses unveiled that H3.5 predominantly localized to the nuclei of primordial sperm cells, in particular spermatocytes (Fig. 3). In stark contrast, spermatids nuclei exhibited no H3.5 signals, consistent with the replacement of most nucleosomes by protamines. Additionally, spermatogonia, Sertoli cell nuclei and Leydig cells displayed very weak or no H3.5 signals (Fig. 3 and Supplementary Figure S1 [zoomed images]).

### Elevated H3.5 expression in seminomas

Our investigation extended to evaluate the hypothetical role of H3.5 in testicular pathologies: Although male germ cell tumors are relatively rare (1.5% of all cancers), they are the most common tumor between the ages of 20 and 45. The pathomechanistic development of a germ cell tumor is caused by the defective maturation of the primordial germ cells into pre-spermatogonia. This leads to polyploidization. The germ cell transformed in this way represents the in situ germ cell neoplasia, which remains dormant until puberty. Usually only after the onset of puberty can the formation of seminomas and non-seminomas occur. This tumor progression is caused by the gain or loss of chromosomal regions (e.g. overexpression of p53, detection of excess copies of the isochromosome i(12p), loss of c-Kit expression and deregulation of the cell cycle at the G1/S checkpoint)^25–27^. Whether and to what extent supernumerary 12p copies could influence the expression of H3-5 gene (H3F3C) on 12p11.21 is not yet known.

**Fig. 4:**
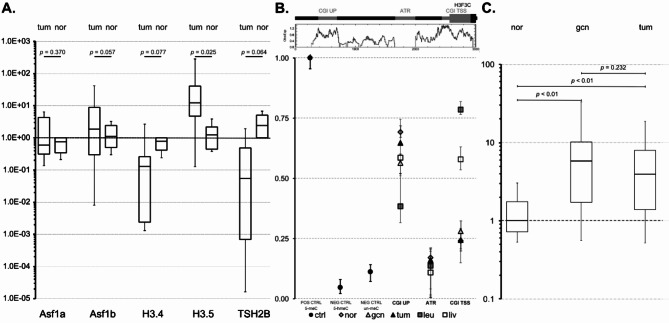
(**A**) Box plot diagram of the expression analysis. The expression of the genes Asf1a, Asf1b, H2A.Bbd, H3.4, H3.5, and TSH2B (x-axis) in tumor tissue (tum) and normal testis (nor), normalized to ACTB, GAPDH and RPS27 (y-axis), is depicted. (**B**) DNA of leukocytes, liver cells, normal testicular cells, germ cell neoplasia in situ (gcn) cells and testicular tumor cells (Seminoma) exhibited overlapping amounts of intermediately methylated DNA at the distant CpG island. In contrast, the CpG island adjacent and overlapping to H3-5 exhibited hypermethylation in DNA from leukocytes and liver cells. In contrast, the same CpG island exhibited hypomethylated DNA in normal and tumor testicular cells and CIS. DNA methylation levels within the AT-rich segment were almost indistinguishable from unmethylated and hydroxymethylated control DNA. These observations suggest that DNA methylation of the CpG island adjacent and overlapping with H3-5 could play a role in the tissue-specific differential regulation of H3.5 expression. However, DNA methylation did not seem to be involved in the elevation of H3.5 expression in a fraction of seminoma-type testicular tumors. (**C**) Boxplots of gene copy number results. Graphical representation of the fold changes of H3F3C in the three examined tissue types. nor = healthy testicular tissue, gcn = germ cell neoplasia in situ, tum = testicular tumor tissue.

Using qRT-PCR, we analyzed the expression of H3.5 mRNA purified from 3 donors of normal testis and 16 seminoma specimens (Fig. 4). For normalization, we utilized 3 house keeping genes, i.e. actin beta (ACTB), glyceraldehyde-3-phosphate dehydrogenase (GAPDH) and ribosomal protein S27 (RPS27). For comparison, we assesses the expression of the histone H4-H3 chaperones Asf1a and Asf1b and two other testis-specific histone variants, H3.4 and TSH2B.

Interestingly, we observed elevated H3.5 mRNA expression in seminoma specimens, compared to samples from 3 normal testis donors. The observed median fold change increase of H3.5 mRNA in seminomas vs. normal testis was associated with *p* = .025 (Fig. 4A). Notably, the inspection of individual’s qPCR data revealed that H3.5 mRNA expression was elevated in the majority of individuals, but not in all. Whereas we saw no difference in the expression of Asf1a and Asf1b mRNAs, we see, cautiously interpreted, a slight trend of downregulation of H3.4 (*p* = .077) and TSH2B (*p* = .064) in seminaries versus normal testes. This observation prompted an exploration of the potential molecular reasons for differential regulation of H3.5 in seminomas.

### DNA methylation patterns in testicular cells

Hypothesizing that DNA CpG methylation could be involved in the differential regulation of H3-5 gene expression, we utilized methylated-DNA immunoprecipitation (MeDIP) in combination with qPCR to assess DNA CpG methylation at three specific positions: 1. In a distant CpG island upstream (CGI up) of the H3.5 gene (H3-5 alias H3F3C); 2. in a CpG island that overlaps the H3-5 promoter, the TSS as well as the 5’-end of the gene body (CGI TSS); 3. an AT-rich sequence (ATR) segment between both CpG islands (Fig. 4B). For control and calibration, we used totally methylated (5-meC) DNA, hydroxymethylated (5-hmeC) DNA as well as unmethylated (un-meC) DNA. Here, we define DNA methylation levels within the lowest tercile as “hypomethylated”, within the middle tercile as “intermediate methylation” and within the highest tercile as “hypermethylated”, whereby the totally methylated DNA (5-meC) reference was set 1.0 (100%).

We detected only little variance in DNA methylation in the more distant ‘CGI up’ and in the ‘ATR’ between the different samples. The DNA methylation at ‘CGI up’ was mostly at the level of intermediate methylation and hypomethylated at the ATR.

Significant differences only occurred at ‘CGI TSS’, namely in the comparison between tissue samples without H3-5 gene expression (liver and leukocytes) and testis, regardless of whether the testicular samples originated from tumor tissue or normal tissue: ‘CGI TSS’ was intermediate or hypermethylated in leukocytes and liver, but it was hypomethylated in normal testis or seminomas (Fig. 4B). These observations suggest a role for DNA methylation in the tissue-specific regulation of H3.5 expression. Interestingly, DNA methylation did not seem to contribute to the elevation of H3.5 expression in some seminoma-type testicular tumors.

### H3-5 copy number gain and seminoma specimens

We hypothesized that in seminomas H3-5 copy number gain could be a contributing factor in H3.5 mRNA expression, since H3-5 is situated in a genomic region of the short arm of chromosome 12 (12p11-p13) whose amplification is often associated with seminomas, whereby H3-5 is adjacent^28^. Although this hypothesis cannot be applied to all cases, because H3-5 is apparently not part of the shortest region of overlap of amplification (SROA), it could apply to some seminoma cases in which the amplification affects a more extensive region. To investigate this possibility, we applied qPCR and utilized the GAPDH gene for normalization, which is located in relative proximity to the telomere of 12p far outside of the SROA (12p13.31). The elevated median copy number of H3-5 in comparison to GAPDH suggests the presence of 12p gain in both germ cell neoplasia in situ (GCNIS) (median fold change = 5.79) and in seminoma tumor samples (median fold change = 3.93), when normal testicular samples (*n* = 3) were compared with GCNIS (*n* = 10) and seminomas (*n* = 10) (Fig. 4C).

### Protein-level regulation through histone chaperones

**Fig. 5:**
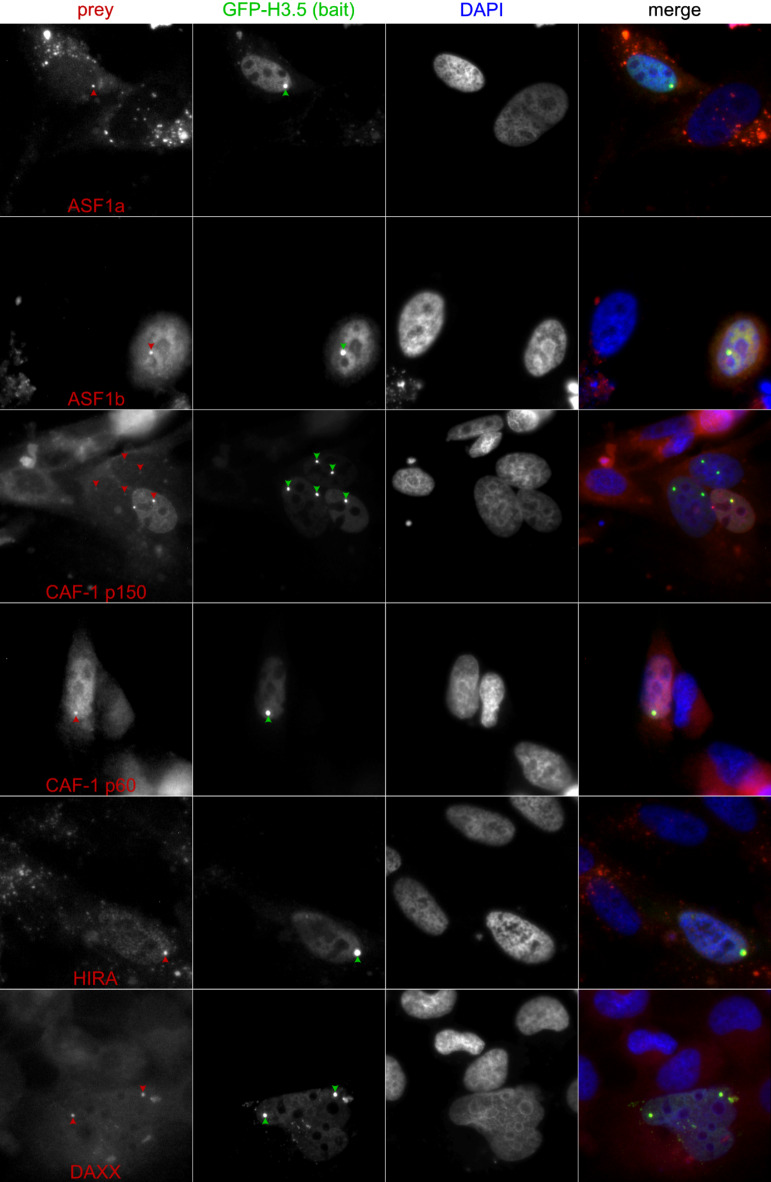
To understand, how H3.5 is regulated by interactions with histone chaperones on the protein level, we performed fluorescence-2-hybrid assays. We interrogated the nuclear localization, chromatin patterns and interactions of GFP-H3.5 with Asf1, HIRA, CAF as well as DAXX. The latter were fused with RFP. We observed that H3.5 physically interacted with Asf1, HIRA or DAXX, but not with CAF, when interrogated proteins-of-interest were expressed under CMV promoter control in baby hamster kidney (BHK) cells.

In an effort to comprehend the protein-level regulation of H3.5, we conducted fluorescence-2-hybrid assays^29^, probing interactions with histone chaperones Asf1a and Asf1b, HIRA, CAF p150, and DAXX. These experiments were conducted in baby hamster kidney (BHK) cells, which exhibit a GFP-trap in the nucleus. We used the pTagGFP2-C for GFP-H3.5 (bait) expression in combination with different constructs of interest, which allowed the expression of Asf1a and Asf1b, HIRA, CAF p150, and DAXX, respectively, as red fluorescent fusion proteins (prey). We used immunofluorescence microscopy to assess possible H3.5/histone chaperone interactions. Whilst binding of GFP-H3.5 (bait) is enforced in the nuclear GFP-trap, the red fluorescent signals only colocalize with the trap, when there is a physical interaction between GFP-H3.5 and the histone chaperone of interest. Our results unveiled that GFP-H3.5 can physically interacted with all interrogated histone chaperones, Asf1a and Asf1b, HIRA, CAF p150 and DAXX (Fig. 5).

**Fig. 6:**
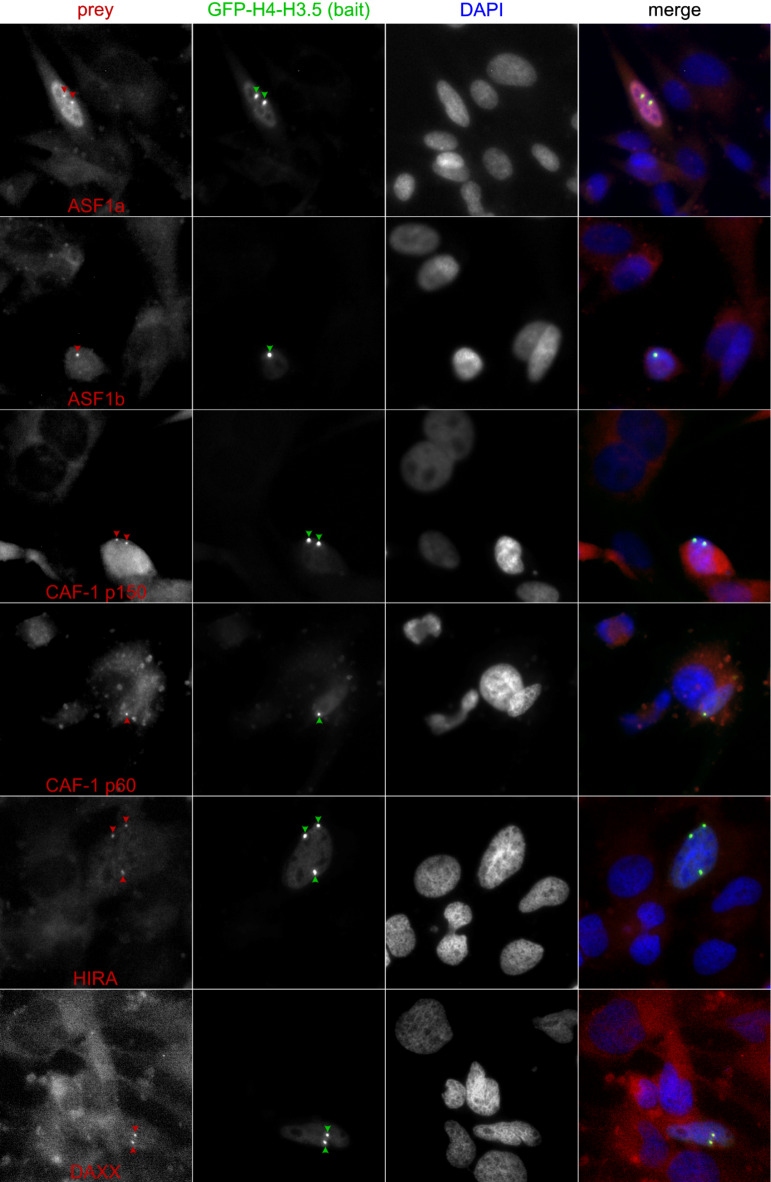
To understand, how H3.5 is regulated by interactions with histone chaperones on the protein level, we performed fluorescence-2-hybrid assays. We interrogated the nuclear localization, chromatin patterns and interactions of GFP-H3.5 with Asf1, HIRA, CAF as well as DAXX. The latter were fused with RFP. We observed that H3.5 physically interacted with Asf1, HIRA or DAXX, but not with CAF, when interrogated proteins-of-interest were expressed under CMV promoter control in baby hamster kidney (BHK) cells.

However, histone H4-H3 dimers rather than monomers are substrates of these histone chaperones. Therefore, in order to control the presence of H4-H3.5 dimers and to complement the above experiments, we constructed pTagGFP2-C constructs which allowed the expression of GFP-H4-H3.5 fusion proteins. This idea was inspired by the natural phenomenon that eukaryote-like histones are encoded as H4-H3 (and H2B-H2A) fusion proteins in the genome of Marseilleviridae. These histones of Marseilleviruses form nucleosomes and are essential for their replication cycle in Acanthamoeba^30^. Using GFP-H4-H3.5 as bait for F2H assays in similar ways as described above, immunofluorescence microscopic analyses showed that GFP-H4-H3.5 can physically interacted with all interrogated histone chaperones, Asf1a and Asf1b, HIRA, CAF p150 and DAXX, strongly confirming the results obtained with GFP-H3.5 above (Fig. 6) and shedding light on its potential role in chromatin dynamics and gene regulation during testicular differentiation.

## Discussion

### Histone H3.5 expression patterns in testicular cell differentiation

The spermatogenesis is a highly coordinated and protracted process occurring over several weeks to months within the testes. It encompasses a complex sequence of events with checks and balances, involving a comprehensive remodeling of the genome and chromatin reorganization at the level of epigenetic plasticity. Of particular interest is the apparent remarkable diversity of histone variants present in humans and closely related primates, which seem to govern this process. The loss of the majority of nucleosomes and their replacement by protamines in the final stages of spermiogenesis merely marks the concluding, radical step in a series of dynamic processes. These processes entail the exchange of specialized histone variants facilitated by histone chaperones. Despite the crucial significance of these molecular details for spermatogenesis, they remain largely veiled in obscurity to date. Further research is required to gain a deeper understanding of the underlying mechanisms.

Within this histone variant ‘orchestra’, hominoid-specific histone H3.5 is a pivotal player in testicular cell differentiation exhibiting intriguing patterns of expression and regulation within the testicular microenvironment. Utilizing c-Kit/CD117 as a separation marker, our investigation unveiled distinct distributions of H3.5 mRNA in testicular cell fractions. Specifically, H3.5 mRNA was enriched in c-Kit/CD117-positive fractions, primarily comprising primordial spermatogonia and mature sperm cells, while c-Kit/CD117-negative fractions were enriched with Sertoli and Leydig cells. This fractionation highlights the cell type-specific regulation of H3.5 during testicular cell differentiation. However, in our hands the fractionation of testicular cells using c-Kit/CD117 antibodies has proven to be rather crude. Examination of publicly available scRNA-seq data partially explains the limited suitability of c-Kit/CD117 as a marker but significantly refines the picture for H3.5 as well as for many other chromatin proteins, such as histone variants and histone chaperones. H3.5 is clearly expressed in spermatocytes and to a limited extent in spermatids. However, its precise function remains elusive. Structural biologists’ analyses have shown that H3.5 nucleosomes can be comparatively unstable^17,20^, hypothetically indicating intermediate replacement function of H3.5. However, this perspective is greatly constrained by the stoichiometric excess of H3.3 at every stage of spermatogenesis.

### Insights from antibody development and immunofluorescence analysis

Further confirmation of H3.5’s role involved the development of specific antibodies and immunofluorescence microscopy. Our results demonstrated the enrichment of H3.5 in the nuclei of primordial sperm cells, with negligible signals in mature sperm cells. Additionally, Sertoli and Leydig cell nuclei exhibited weak or no H3.5 signals, emphasizing the cell type-specific regulation of H3.5 during testicular differentiation.

### Implications in testicular pathologies: elevated H3.5 expression in seminomas

Elevated H3.5 mRNA expression was observed in seminoma specimens, suggesting a potential role for H3.5 in testicular pathologies. DNA methylation studies revealed differential methylation patterns at key loci, implicating the CpG island overlapping the H3-5 promoter in tissue-specific regulation. Interestingly, DNA methylation did not seem to be involved in the elevation of H3.5 expression in some seminoma-type testicular tumors.

### Corroborative evidence from copy number analysis

Investigation into H3-5 copy number gain, frequently associated with seminomas, provided corroborative evidence for elevated H3.5 expression in these tumors. This observation was consistent across carcinoma in situ (CIS) and tumor tissue samples, aligning with known clinical associations.

### Protein-level regulation through interaction assays

We believe that we can gradually unravel the function of H3.5 (and other histone variants) by better understanding the differential interplay and interactions with histone chaperones. To this end, we have taken initial important steps through F2H analyses. Exploring the molecular regulation of H3.5, fluorescence-2-hybrid assays revealed physical interactions between H3.5 and histone chaperones Asf1, HIRA, and DAXX, suggesting potential roles in chromatin remodeling and gene regulation during testicular differentiation.

### Limitations and future directions

While our study provides valuable insights, several limitations warrant consideration. Cell fractionation and c-Kit/CD117 as a marker may have inherent limitations, impacting the purity of cell fractions. Antibody specificity tests were done via Western blot experiments. Results suggests that H3.5 could be distinguished from certain H3 variants under specific conditions. However, the results of immunofluorescence assays must be interpreted with care: Although the immunofluorescence microscopy analyses seem to be in good agreement with the H3.5 expression observed in single-cell RNA-seq data, cross-reactivity of anti-H3.5 antibodies with other H3 variants cannot be completely ruled out due to their extremely conserved primary structure. This is particularly the case because RNA-seq data suggest that H3.5 is quantitatively underrepresented, especially when compared to H3.3. Moreover, all results from analyses on patients’ samples have preliminary, observational character. We therefore explicitly state that the present study does not aim to meet the standards of a clinical trial and should only be interpreted as an observational, hypothesizing pilot study. Larger sample sizes and mechanistic studies are needed to establish causality and address potential confounding factors.

In conclusion, our findings elucidate the complex interplay of H3.5 in testicular cell differentiation and its involvement in testicular pathologies. Addressing limitations and conducting further research will enhance our understanding of male reproduction and may offer insights into the etiology and progression of testicular diseases.

### Methods

#### Samples

Research involving human research participants were performed in accordance with the Declaration of Helsinki and with approval of the Witten/Herdecke University Ethics board (No’s. 90/2011 and 138/2013). In addition, for investigations involving human subjects, informed consent has been obtained from all participants.We analyzed a cohort of 16 testicular tissue samples that exhibited malignant characteristics. These samples were obtained as part of therapeutic orchiectomies performed on male patients suspected or diagnosed with intrascrotal tumors at the Department of Urology and Pediatric Urology of HELIOS University Hospital Wuppertal between 2010 and 2013. Following diagnostic processing at the Institute of Pathology and Molecular Pathology of HELIOS University Hospital Wuppertal, these tissue samples, with the consent of the patients, were made available to the research laboratory of the Center for Clinical Medicine Research (ZFKM), HELIOS University Hospital Wuppertal. The tissue samples consisted of approximately 100 mg biopsies originating from the central tumor area, as well as preparations from tumor-adjacent tissue. The pathological entities within the samples comprised nine classic seminomas, three cases of embryonal carcinoma each, and three cases of mixed-differentiated malignant germ cell tumors. Additionally, one case was diagnosed as a liposarcoma of the spermatic cord. The age of the patients in the cohort ranged from 27 to 86 years, with a mean age of 42.8 years and a median age of 36.5 years (25th percentile: 31.75 years, 75th percentile: 47.75 years). Furthermore, we examined three tissue samples from healthy testicular tissue. These samples were obtained from patients who underwent orchiectomy as part of the treatment for metastatic prostate cancer. In addition to these tissue samples, we used leukocytes and liver tissue. All tissue samples were preserved in RNAlater immediately after collection and stored at -80 °C. The analysis of all collected samples was conducted between August 2013 and March 2014, with the informed written consent of all patients.

#### Pachytene spermatocyte separation using c-Kit/CD117 as a separation marker

For enrichment of CD117-positive cells from human testicular tissue, the method commenced with the thawing of the tissue, followed by meticulous dissection in 1 ml phosphate-buffered saline (PBS) using forceps within a Petri dish. Subsequently, enzymatic digestion was undertaken by supplementing the tissue fragments with a solution containing 1 mg/ml Hyaluronidase Type II (minimum 500 U/mg) and 1 mg/ml Collagenase Type IV, followed by an incubation period of 1 h at 37 °C. The enzymatically digested tissue was then mechanically dissociated through a 70 μm cell strainer using a stamp (syringe) and collected in a 50 ml Falcon tube, subsequently replenished with PBS to a final volume of 20 ml. The resultant suspension underwent centrifugation at 470xg for 8 min, with the subsequent removal of the supernatant. Erythrocyte lysis ensued through resuspension of the pellet in 1 ml Solution 1, followed by a 1-minute incubation period. This was succeeded by the addition of 1 ml Solution 2 and 8 ml PBS, and the suspension was passed through a cell strainer once again before undergoing an additional 8-minute centrifugation at 470xg. The resulting pellet was resuspended in 1 ml PBS, and cell counting was performed using a Neubauer counting chamber. Subsequently, the CD117 MicroBead Kit from Miltenyi Biotec was employed. The cells were adjusted to a total cell density of 10^8 cells per 300 µl buffer, and 100 µl of FcR Blocking Reagent and 100 µl of CD117 MicroBeads were added per 10^8 total cells. Following thorough mixing, the cell suspension was incubated for 15 min at 4 °C. The cells were then washed with 1–2 ml PBS per 10^8 total cells, centrifuged at 470xg for 8 min, and the supernatant discarded. Up to 10^8 cells were resuspended in 500 µl PBS. Magnetic-activated cell sorting (MACS) was performed by placing the LS column in the magnetic field of the MACS Separator. The column was prepared with 3 ml PBS, and the cell suspension was applied to the column. The eluate, containing unmarked cells, was collected in a tube. The column was subsequently washed with 3 × 3 ml PBS, and the remaining cells were eluted into a separate tube. Following removal of the magnet, the column was placed over a new tube, and 5 ml PBS was used to flush the cells from the column by applying pressure with a plunger. The eluate, comprising CD117-positive cells, was collected. The cells from both fractions were deemed suitable for immediate RNA isolation or could be stored at -80 °C for future analyses.

#### Nucleic acids

For RNA extraction, 3 × 3 mm tissue samples were isolated from testicular tissue stored at -80 °C and frozen in liquid nitrogen. Subsequently, the samples were pulverized using a Cellcrusher. The powdered samples were homogenized in 1 ml of TRIZOL^®^ and shaken for 15 s. To separate the various nucleic acids, 200 µl of chloroform was added and mixed using a vortex device. After a 2-minute incubation at room temperature, the sample was centrifuged at 13,000 rpm for 2 min in a 1.5 ml tube. The centrifugation resulted in the distinction of the lower red organic phase, the whitish DNA phase, and the colorless aqueous RNA phase. The aqueous phase was transferred to a new reaction vessel, mixed with 550 µl isopropanol by pipetting up and down, and precipitated at -20 °C overnight. For further purification, the sample was centrifuged at 13,000 rpm and 4 °C for 45 min. The supernatant was discarded, and the pellet was overlaid with 500 µl of ice-cold 75% ethanol. After 15 min of centrifugation, the supernatant was decanted, and the pellet was air-dried with the tube lid open at 55 °C. The gel-like pellet was dissolved in 40 µl of RNase-free water and immediately frozen at -80 °C. RNA quality and quantity were assessed by microcapillary electrophoresis using an Agilent Bioanalyzer 2100 similarly as described^31^. cDNA synthesis was performed using the QuantiTect Reverse Transcription Kit (Qiagen) according to the manufacturer’s instructions. The protocol called for 1 µg of mRNA, so the volume of mRNA to be used was calculated based on NanoPhotometer^®^ measurements, ranging from 1 to 5 µl. The mRNA stored at -80 °C was thawed on ice, and to remove any remaining genomic DNA, it was mixed with wipeout buffer (2 µl), containing DNAse 1, and RNase-free water, bringing the total volume to 14 µl. This mixture was incubated at 42 °C for 10 min.

For the master mix of the actual mRNA to cDNA transcription, 1 µl of reverse transcriptase, 1 µl of Primer-Mix, and 4 µl of the corresponding buffer were combined with the previous reaction mixture, resulting in a total volume of 20 µl. The samples were then incubated at 42 °C for 30 min, and the reaction was stopped by a final 3-minute incubation at 95 °C. The cDNA was subsequently stored at -20 °C or used directly for gene expression analysis via quantitative real-time PCR.

Genomic DNA extraction from the tissue samples was carried out using the Genomic DNA-Tissue MiniPrep Kit from Zymo Research. In the first step, tumor-adjacent and tumor-distant tissue were mechanically pulverized to a powder form and then mixed in a 1.5 ml reaction vessel with 95 µl distilled water, 95 µl 2x Digestion Buffer, and 10 µl Proteinase K. The mixture was incubated at 55 °C overnight. After adding 700 µl Genomic Lysis Buffer, the sample was thoroughly mixed by vigorous vortexing and centrifuged at 13,000 rpm for one minute. The supernatant was transferred to a Zymo-Spin™ Column, placed in a Collection Tube, and centrifuged again (1 min, 13,000 g). Subsequently, 200 µl of DNA Pre-Wash Buffer was added to the Zymo-Spin™ Column, and the sample was centrifuged in a new Collection Tube. For the final washing step, 400 µl g-DNA Wash Buffer was added to the Zymo-Spin™ Column. After centrifugation (1 min, 13,000 rpm), the DNA was transferred to a 1.5 ml reaction vessel by decanting. The DNA was washed from the Zymo-Spin™ Column by adding 50 µl distilled water after a 10-minute incubation at room temperature and a final centrifugation for 30 s at 13,000 rpm. The DNA was then stored at -20 °C.

#### Real time PCR

A list of oligonucleotides used is provided as appendix (Supplementary Table 1). Quantitative measurement of mRNAs was analyzed from cDNA libraries (as described in^31^) using primer pairs as listed in Supplementary Tables 1 and the QuantiTect SYBR Green PCR Kit (Qiagen) using a Corbett Rotor-Gene 6000 qPCR machine. PCR conditions were as follows: 95 °C for 15 min, 40 cycles of (95 °C for 15 s, 60 °C for 30 s). Amplicon melting was performed using a temperature gradient from 55 to 95 °C, rising in 0.5 °C increments. For relative comparative quantification of quantitative expression fold changes we utilized the ΔΔCt method^32^ using genes beta actin (ACTB), glyceraldehyde-3-phosphate dehydrogenase (GAPDH), ornithine decarboxylase antizyme 1 (OAZ1), ribosomal Protein L19 (RPL19) and ribosomal Protein S27 (RPS27) for normalization.

#### Anti-H3.5 polyclonal antibodies

A polyclonal anti-H3.5 antibody was developed by Diagenode (Seraing, BEL) based on a synthetically produced peptide sequence (RKSTPSTCGVKPHR, corresponding to H3R27-R40). Two rabbits were immunized with this peptide over a period of 4.5 months, with blood samples collected at various intervals for serum isolation. Enzyme-linked Immunosorbent Assays (ELISAs) were conducted to assess the antibody response, confirming recognition of the peptide. Further analysis indicated successful immunization in one rabbit (A1578) while showing limited response in another (A1577) (Supplemental information 1). Affinity chromatography was then employed to purify the antibody from crude serum, yielding a specific antibody preparation suitable for subsequent assays.

#### Cell culture and fluorescence-two-hybrid (F2H) assays

The Fluorescent Two-Hybrid (F2H) Assay (Chromotek, Martinsried, Germany) is a technique used to investigate protein-protein interactions within mammalian cells in vitro. This assay relies on lipofection, a method of introducing nucleotides into cells using Lipofectamine 3000. Initially, cells are seeded and then transfected with plasmids containing genes for GFP- and different red fluorescence-fusion proteins. These proteins act as bait and prey, respectively, and are designed to fluoresce green and orange/red, respectively. Following transfection, the cells are incubated 12 h to allow for protein expression and interaction. Within the nucleus of the transfected cells, a GFP-binding ‘trap’ is present, enabling the localization of GFP-fusion proteins. Interaction between the GFP-bait and the red fluorescent prey leads to the colocalization of green and red fluorescence signals at the GFP-binding ‘trap’. This colocalization is detectable using fluorescence microscopy. If no protein interaction occurs, only the green fluorescence signal from the GFP-bait protein is observed within the nucleus, while nonspecific red staining may be visible throughout the cell.

#### Immunofluorescence microscopy

Sections of seminiferous tubules were fixed for 10 min in 2% PFA/DPBS, washed twice with DPBS and then permeabilized using 0.5% Triton X-100/DPBS, followed by 0.1 N HCl for exactly 5 min for antigen retrieval and successive washes with DPBS. Blocking was done in 3% BSA/0.1% Triton X100/DPBS. Subsequently, the primary (H3.5/panH3) antibodies were incubated in blocking buffer for 1 h/37°C: 1. polyclonal rabbit anti-H3.5 at 1:100, polyclonal rabbit panH3 at 1:100. Then polyclonal goat anti-rabbit IgG (H + L) cross-adsorbed secondary antibody Alexa Fluor 488 (ThermoFisher) was applied at 1:100 dilution. Eventually, SYTOX™ Orange Nucleic Acid Stain was used for DNA counterstaining at 0.1 µg/mL followed by washes and mounting with Prolong Gold (Invitrogen). Sections were analyzed via confocal laser scanning microscopy (CLSM) using a Zeiss LSM 5 Pa microscope with a water objective lens (Plan-Neofluar 25/0.8). Fluorochromes were visualized using an argon laser with excitation wavelengths of 488 nm and 543 nm. Images were scanned sequentially, generating 8-bit grayscale images with a resolution of 512 × 512 pixels. Each section image was averaged from four scans to improve signal-to-noise ratio. The grayscale images were overlaid into RGB images and assembled into tables using ImageJ and Affinity Photo Software.

Alternatively, baby hamster kidney (BHK) cells were grown for F2H assays on coverslips in 6-well plates. F2H preparations were conducted as described above. Eventually, DAPI was used for DNA counterstaining at 0.1 µg/mL followed by washes and mounting with Prolong Gold (Invitrogen). Acquisition of images was done with a Olympus CKX53 microscope and a Hamamatsu ORCA-spark digital CMOS camera. Fluorochrome images (channels: red, green, blue) were acquired sequentially generating 8-bit grayscale images. The 8-bit grayscale single channel images were overlaid to an RGB image assigning a false color to each channel using open source software ImageJ (NIH Image, U.S. National Institutes of Health, Bethesda, Maryland, USA, https://imagej.nih.gov/ij/).

#### DNA methylation analyses by MeDIP-qPCR

CpG islands near the H3F3C promoter region were identified using the EMBOSS CPG-plot tool. Complementary primers were designed for PCR amplification, and the CpG islands, spanning a length of 3000 bp downstream of the transcription start site, were examined for their methylation patterns.

The MeDIP experiments in this study were conducted using the MagMeDIP Kit (Diagnode) following the manufacturer’s instructions. Briefly, genomic DNA was isolated from liver tissue, leukocytes, testicular tumor tissue, and adjacent testicular tissue from two selected patients. Subsequently, DNA shearing was performed for the six samples, where each sample was diluted to a concentration of 0.1 µg/ml with GenDNA TE buffer (300 µl–30 µg per sample). DNA fragmentation was accomplished using the Bioruptor (Diagenode) with the following protocol: 10 cycles of 15 s on and 15 s off. The magnetic beads were cleaned according to the protocol, and the incubation mix for immunoprecipitation was prepared. A simple version of the incubation mix with controls was chosen, consisting of various reagents as listed. The mix was incubated at 95 °C for three minutes, rapidly cooled on ice, and briefly centrifuged at 4 °C. In addition to the IP reaction setups, aliquots for the corresponding controls were prepared, each containing 20% of the incubation mix volume. Next, the antibody mix was prepared and added to the incubation mix, along with the cleaned magnetic beads. The samples were then incubated overnight at 4 °C on a rotating wheel. All subsequent washing steps were performed on ice. The tubes were centrifuged and placed on a magnetic rack for one minute. After decanting the supernatant, the samples were washed three times with 100 µl of cold MagWash Buffer-1. Finally, the magnetic beads were washed with MagWash Buffer-2, and the samples were ready for the next step: DNA isolation. For DNA isolation, 100 µl of DNA Isolation Buffer (DIB) was mixed with 1 µl of Proteinase K. The immunoprecipitation mix was removed from the magnetic rack, resuspended with 100 µl of DIB, and transferred to new 1.5 ml tubes. The control samples were mixed with 92.5 µl of DIB, and both samples were incubated for 15 min at 55 °C. After an additional 15-minute incubation at 100 °C, the samples were centrifuged at 4 °C and 14,000 rpm for 5 min. The supernatant contained the isolated DNA, which was aliquoted and prepared for quantitative real-time polymerase chain reaction or stored at -20 °C.

#### Analyses of differential gene expression and 12p gain

The Delta-Delta-Ct method was employed for both gene expression analysis and the analysis of 12p copy number. Initially, the Ct value (cycle threshold) for each sample was determined, representing the cycle at which the fluorescence signal first surpasses the threshold of fluorescence background noise. This marks the beginning of the exponential DNA amplification phase. Subsequently, the expression of the target gene was normalized to the expression of an unregulated, ubiquitously expressed reference gene (Housekeeping gene). Following this normalization, the Delta-Delta-Ct value was calculated by subtracting the Delta-Ct value of the control sample (corresponding to healthy testicular tissue) from the Delta-Ct value of the altered tissue (tumor tissue or ICGNU). This Delta-Delta-Ct value was then applied to the equation for n-fold expression, yielding the relative expression difference (Ratio) of the gene between the tumor/ICGNU and healthy testicular tissue.

#### Statement on methodological limitations

The methodological limitations of this study include variability in sample sources, with tissue samples obtained from therapeutic orchiectomies on male patients with suspected or diagnosed intrascrotal tumors, potentially introducing heterogeneity in pathological entities and patient characteristics. Moreover, the small sample size of the cohort (*n* = 16) may limit the generalizability of findings and statistical power. Additionally, the wide age range of patients (27 to 86 years) poses challenges related to age-related changes in gene expression and tissue characteristics. The control tissue, sourced from patients undergoing orchiectomy for metastatic prostate cancer, may not be ideally matched for gene expression studies in testicular tumors. Variability in RNA extraction efficiency and the use of housekeeping genes for normalization in PCR analyses could introduce bias. Despite successful antibody development and purification, potential cross-reactivity of the anti-H3.5 antibody with other histone variants or proteins requires cautious interpretation of results. Furthermore, cell culture-based assays may introduce artifacts, impacting the reliability of protein-protein interaction data interpretation.

## Electronic Supplementary Material

Below is the link to the electronic supplementary material.

Supplementary Material 1

Supplementary Material 2

Supplementary Material 3

Supplementary Material 4

## Data Availability

Data generated in the course of our study are included within the manuscripts.

## Abbreviations

BHK: Baby hamster kidney
BSA: Bovine Serum Albumin
c-Kit: C-Kit protooncogene
cDNA: Complementary DNA
CD117: Cluster of Differentiation 117
CGI: CpG Island
DPBS: Dulbecco’s Phosphate Buffered Saline
ELISA: Enzyme-linked Immunosorbent Assay
EMBOSS: European Molecular Biology Open Software Suite
F2H: Fluorescence-2-hybrid
g-DNA: Genomic DNA
GCNIS: Germ Cell Neoplasia In Situ
GFP: Green Fluorescent Protein
IP: Immunoprecipitation
MACS: Magnetic-activated cell sorting
MeDIP: Methylated DNA Immunoprecipitation
NASP: Nuclear Autoantigenic Sperm Protein
PBS: Phosphate-buffered saline
PFA: Paraformaldehyde
PRM1: Protamine 1
PRM2: Protamine 2
PRM3: Protamine 3
PTMs: Post-translational modifications
qPCR: Quantitative Polymerase Chain Reaction
qRT-PCR: Quantitative Reverse Transcription Polymerase Chain Reaction
RBBP4: Retinoblastoma Binding Protein 4
scRNA-seq: Single-cell RNA sequencing
SROA: Shortest Region of Overlap of Amplification
TNP1: Transition Protein 1
TSS: Transcription Start Site
UBN1: Ubiquitin-60 S Ribosomal Protein L40
5-hmeC: 5-Hydroxymethylcytosine
5-meC: 5-Methylcytosine
un-meC: Unmethylated Cytosine
TE: Tris-EDTA
