# Supplementary figures and images for: Histone variant H3.5 in testicular cell differentiation and its interactions with histone chaperones
Source: Sci Rep. 2024 Dec 19;14:30564. doi: 10.1038/s41598-024-83206-9 (PMC11659419; doi:10.1038/s41598-024-83206-9)

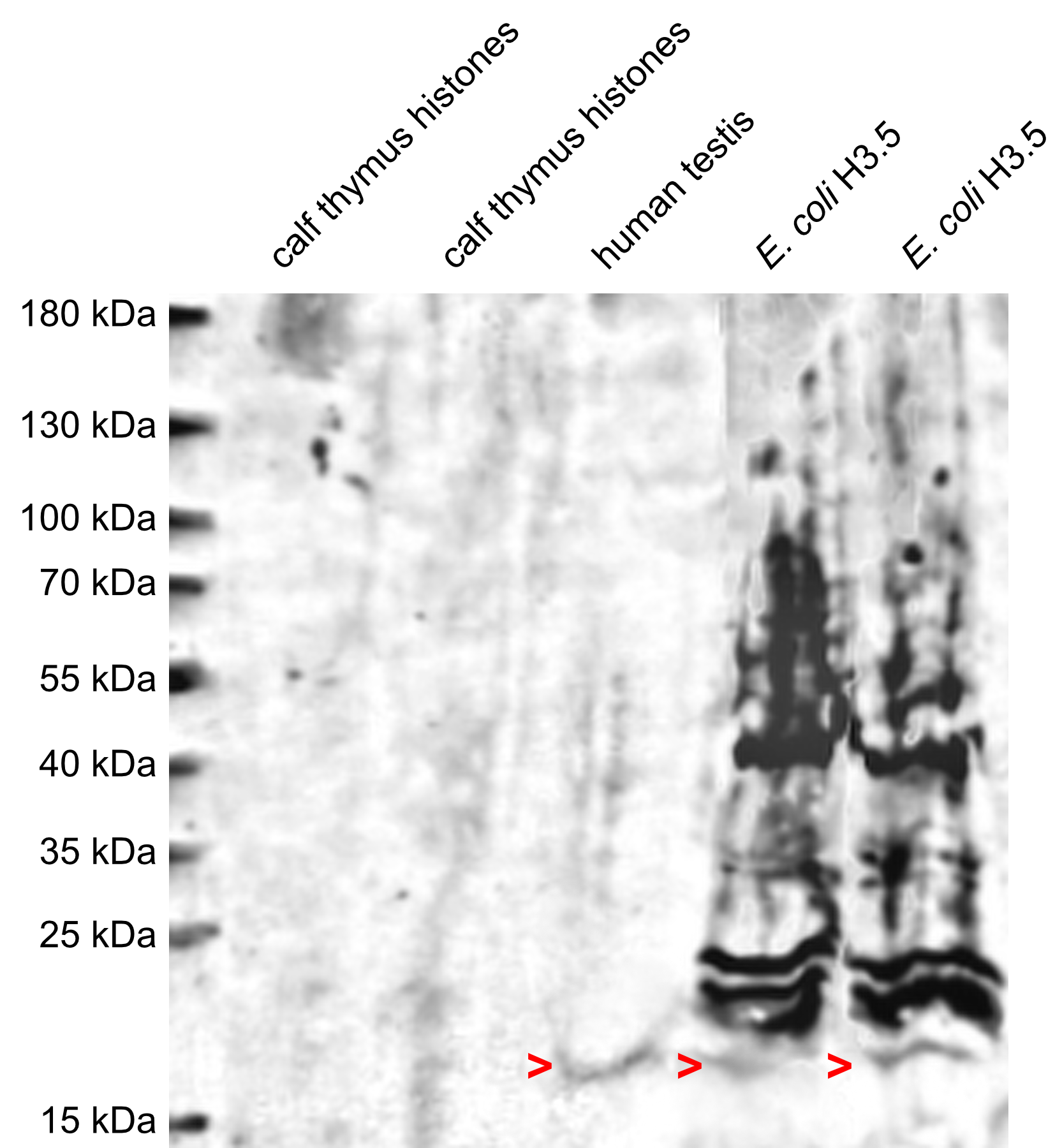

Supplement: Supplementary file 1 — Supplementary Material 1 [file 41598_2024_83206_MOESM1_ESM.tif]
