# Supplementary material for: Histone variant H3.5 in testicular cell differentiation and its interactions with histone chaperones
Source: Sci Rep. 2024 Dec 19;14:30564. doi: 10.1038/s41598-024-83206-9 (PMC11659419; doi:10.1038/s41598-024-83206-9)
Supplement: Supplementary file 2 — Supplementary Material 2 [file 41598_2024_83206_MOESM2_ESM.pdf]

Supplementary Information 1: Production of a Polyclonal anti-H3.5 Antibody

Histone Variant H3.5 in Testicular Cell Differentiation and Its Interactions with Histone Chaperones

Patrick Philipp Weil[1], Anton Pembaur[1], Beatrice Wirth[1], Eda Oetjen[1], Hannes Büsscher[1], Klemens Zirngibl[1], Malte Czarnetzki[1], Stella Braun[1], Jann-Frederik Cremers[2], Daniel Gödde[3], Stephan Degener[4], Jan Postberg[1,*]

*corresponding author

[1-4] See main manuscript for details on authors' affiliations.

To detect H3.5 expression at the protein level, a polyclonal antibody was developed by Diagenode (Seraing, BEL) based on a selected synthetic peptide sequence (#C15 H3.5 variant: RKST-PSTCGVKPHR (H3R27-R40)). This sequence was chosen to exhibit slight but crucial differences from H3.3 (K36del).

Two rabbits (A1577, A1578) were immunized with the synthesized peptide at six time points over a 4.5-month period (Figure SI1_A). Blood was collected from the rabbits at Day 0, 38, 66, 87, and after 4 and 4.5 months to isolate the sera. An Enzyme-linked Immunosorbent Assay (ELISA) was used to assess the animals' immune response to the immunization. ELISAs confirm the antibody's recognition of the peptide used for immunization but do not confirm recognition of the H3.5 protein by the antibody.

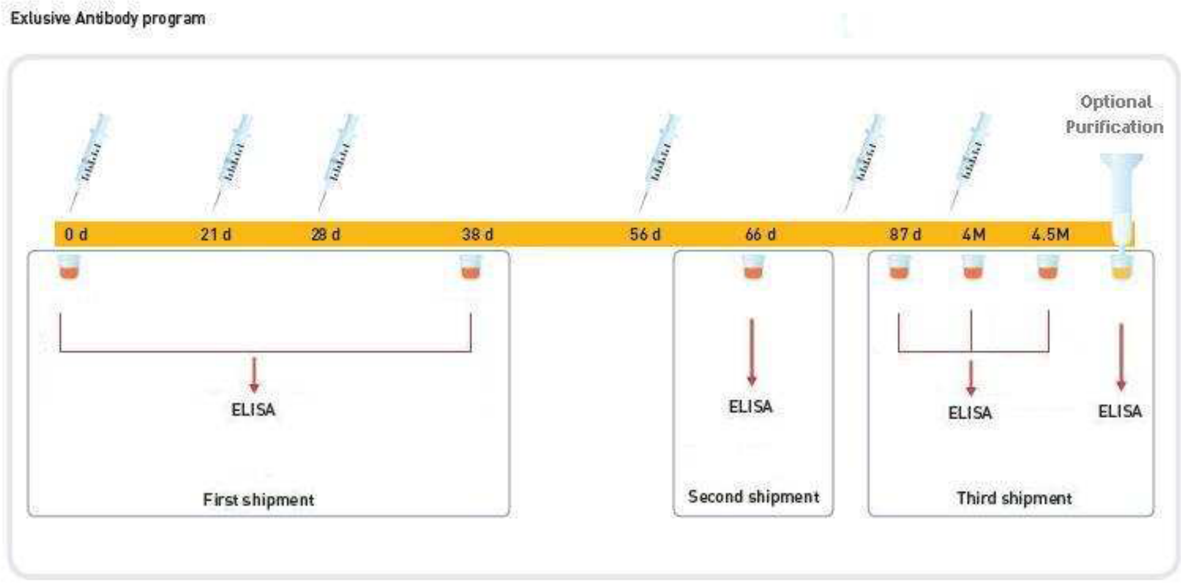

Figure SI1_A. Overview of the production process of the anti-H3.5 antibody by Diagenode (Seraing, BEL). Two rabbits were used for immunization, and blood was collected at Day 0, 38, 66, 87, and after 4 and 4.5 months to determine the antibody titer.

A total of 3 ELISAs were conducted. The first ELISA analyzed sera from days 0 and 38. The antibody detection relies on the optically detectable reaction of a colorimetric substrate. Optical density was measured at various concentrations, and dilutions were plotted logarithmically. Rabbit A1577 showed minimal antibody signals at Day 38, while Rabbit A1578 exhibited clear signals (Figure SI1_B).

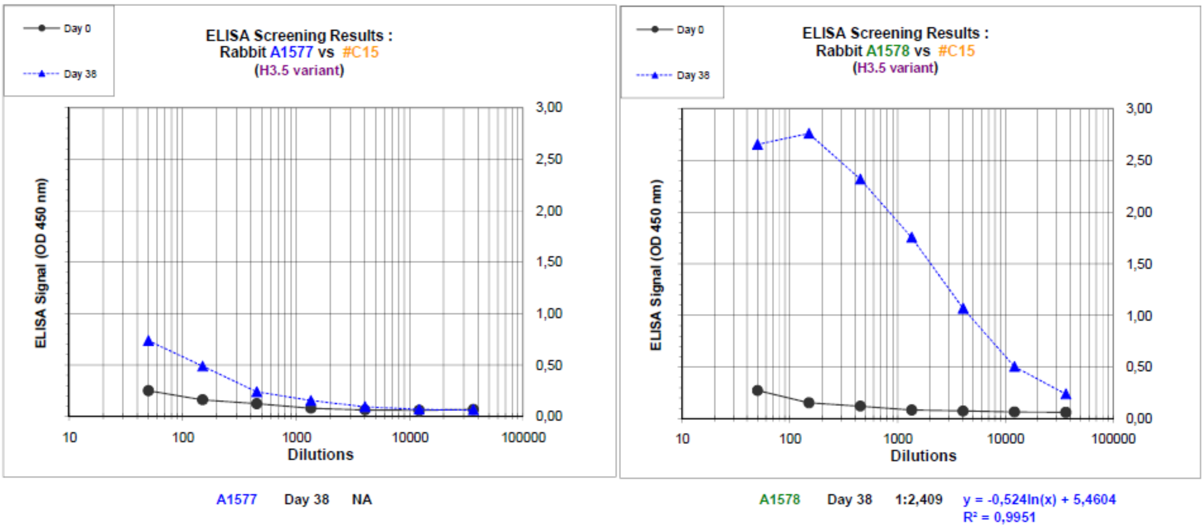

Figure SI1_B. ELISA of sera from Day 0 and 38. The optically detectable reaction of the colorimetric substrate shows clear signals at various dilutions in Rabbit A1578, whereas Rabbit A1577 shows minimal detectable signals.

Another ELISA was performed after 66 days to further investigate immunization based on the measured antibody signals. Sera from Day 0, 38, and 66 were compared. Rabbit A1578 continued to show specific detectable signals, as observed on Day 38. However, signals remained low for Rabbit A1577 (Figure SI1_C).

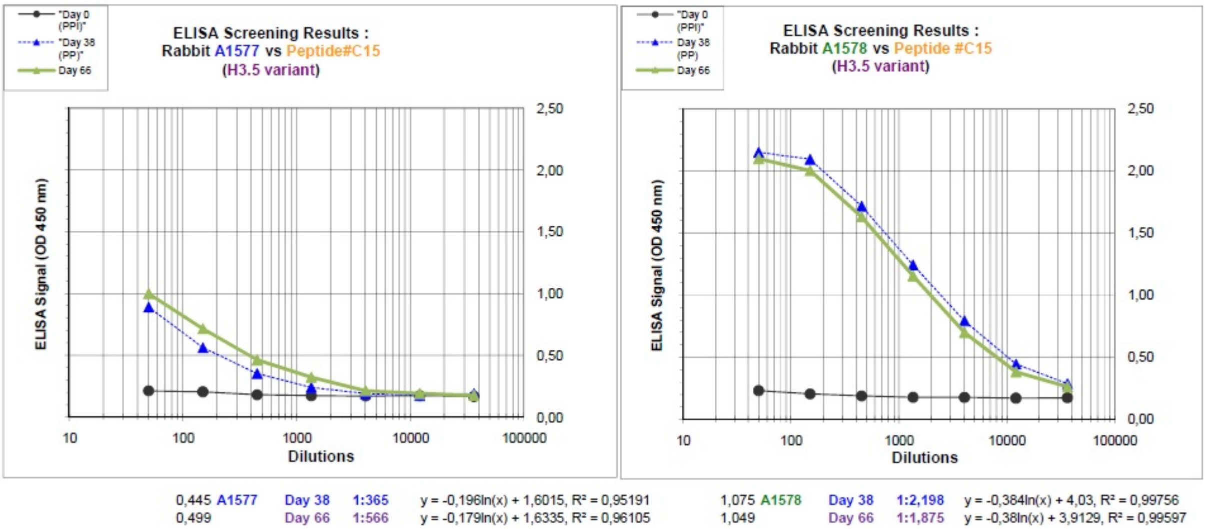

Figure SI1_C. ELISA of sera from Day 0, 38, and 66. The optically detectable reaction of the colorimetric substrate shows clear signals at various dilutions in Rabbit A1578 again, while Rabbit A1577 continues to exhibit minimal detectable signals.

Additional sera were collected at Day 87 and after 4 and 4.5 months and combined with previously collected sera for another ELISA (Figure SI1_D).

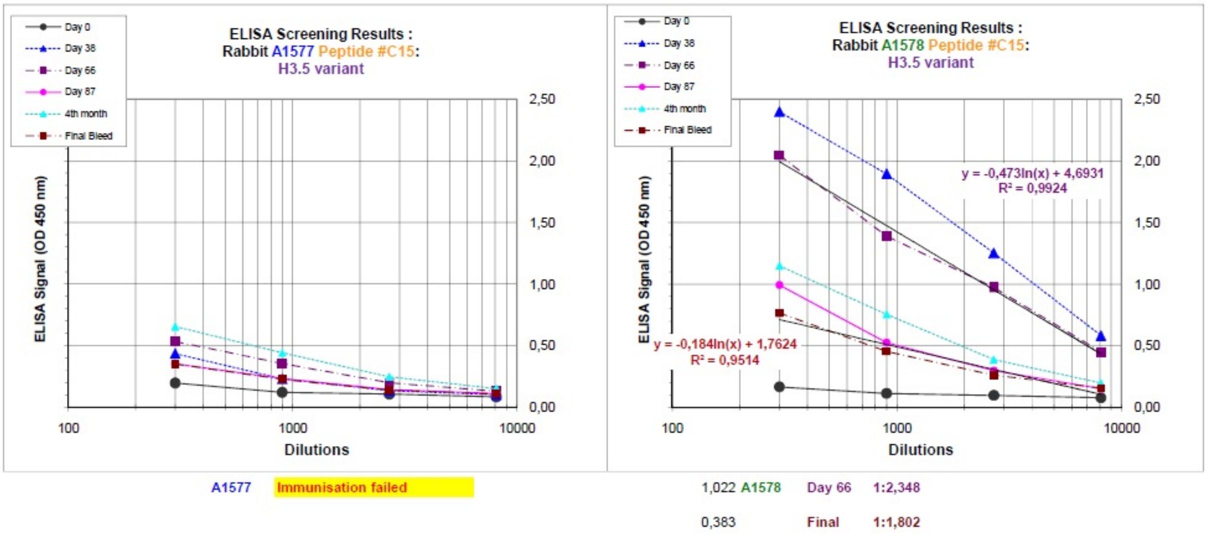

Figure SI1_D. ELISA of sera from Day 0, 38, 66, 87, and after 4 and 4.5 months. Even after 87 days, specific signals at different dilutions are still observable in Rabbit A1578, as well as after 4 and 4.5 months.

Immunization was successful in Rabbit A1578, while Rabbit A1577 showed no detectable signals, indicating failed immunization.

Analysis of sera from Rabbit A1578 continued to show specific detectable optical signals even after 87 days and after 4 and 4.5 months at different dilutions. Immunization with the peptide yielded positive results. Conversely, no signals were detectable in Rabbit A1577, indicating failed immunization. The purified anti-H3.5 antibody was obtained through affinity chromatography against the H3.5 peptide used for immunization from 10 ml of crude serum. To ensure quality, the purified antibody (~0.7 mg/ml), the serum used for purification, and the flow through of the chromatography column were analyzed in another ELISA, with various dilutions (Figure SI1_E).

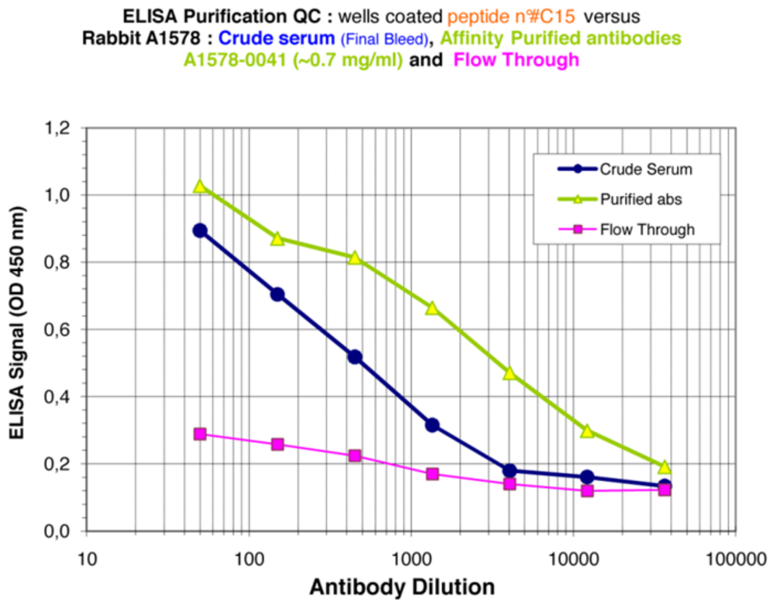

Figure SI1_E. ELISA for quality control of the purified anti-H3.5 antibody. Both the purified antibody (~0.7 mg/ml) and the crude serum used for purification show specific signals compared to the flow through of the chromatography column.

The purified anti-H3.5 antibody was used for investigating H3.5 expression at the protein level via Western analyses (Figure SI2) and via immunofluorescence microscopy using tissue sections of seminiferous tubules (Figure 3).

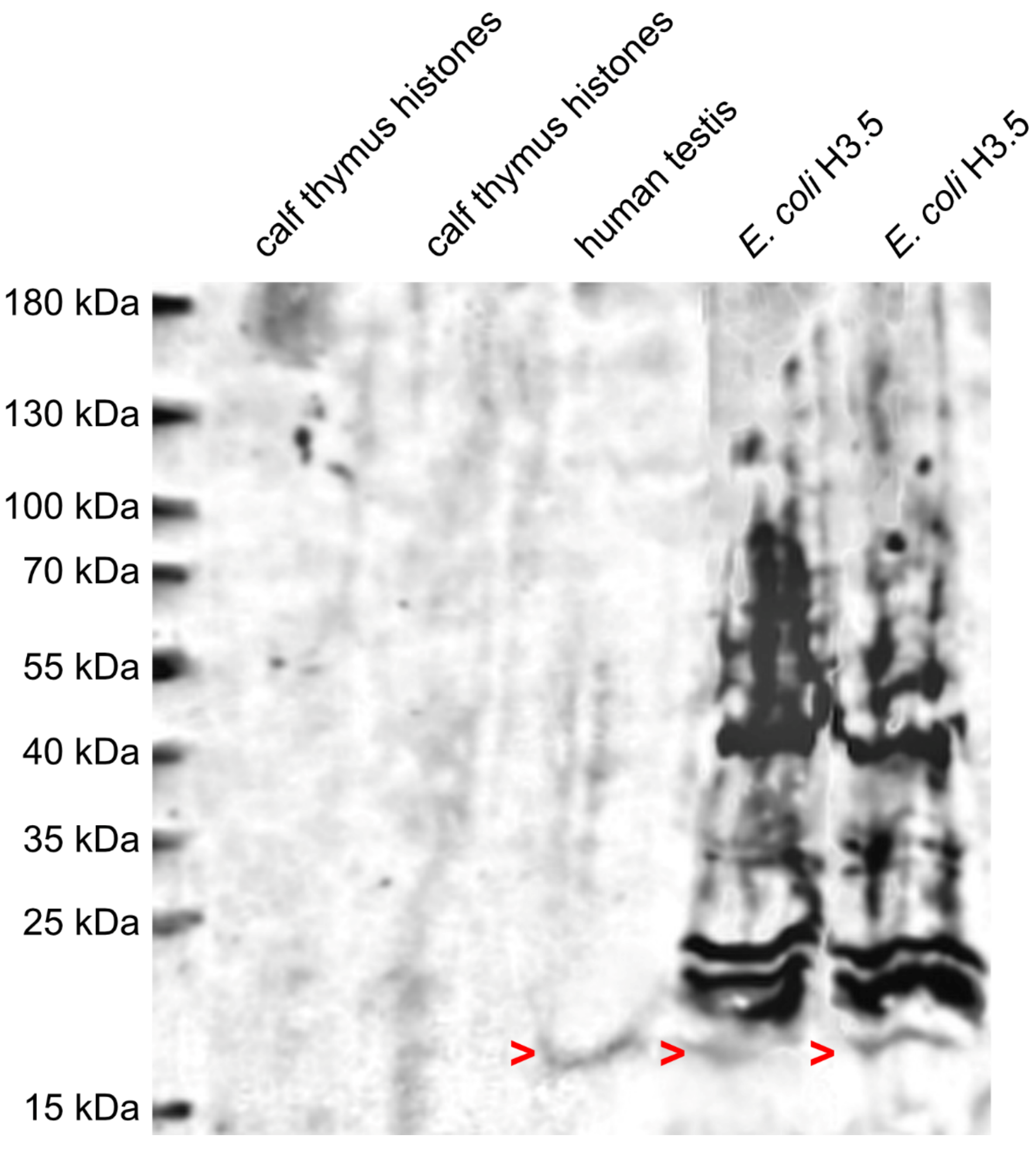

Figure SI1_F. Western analysis using the purified anti-H3.5 antibody. In order to test the specificity of anti-H3.5 antibodies, several protein samples containing H3.5, or histone samples devoid of H3.5 were separated on 4-12% gradient SDS PAGE gels (Serva) and subsequently blotted on nitrocellulose membranes (Amersham). 5% bovine serum albumin (BSA) was used for blocking (Serva). The first two lanes were loaded with a mixture of histones H1, H2A, H2B, H3, and H4, isolated from calf thymus (Roche). Lane 3 was loaded with proteins sampled from normal human testis. Lanes 4 and 5 were loaded with proteins sampled from *E. coli* (BL21/DE3), which were used for the expression of 6-His-H3.5 via

pRSET B (Invitrogen). 3 further lanes were loaded with BSA to achieve a uniform separation. After electrotransfer to a nitrocellulose membrane, the membrane was cut between lanes 4 and 5 into 2 parts for a better fit in incubation containers. To create the final illustration shown above, track 5 was merged with tracks 1-4 as layers using Affinity Photo software (dark color mode). The fading bands of the protein size standard (PageRuler, ThermoFisher) were marked with a pencil. For the final illustration, an image section was chosen that intersects the protein ladder. The upper cut edge runs just above 180 kDa, and the lower cut edge runs just below 15 kDa. The 3 BSA tracks were removed through the right-hand cut edge. A photograph from the lab book showing the unmanipulated nitrocellulose membranes in full dimension was uploaded and proven during the peer review process.

Goat anti-rabbit polyclonal antibodies conjugated with alkaline phosphatase (Jackson Immunoresearch) in combination with BCIP/NBT color development substrate (Promega) were used for detection. Whereas no cross-reaction of anti-H3.5 antibodies was observed in calf thymus histones, a faint but unique approx. 17 kDa band was seen in the human testis sample (red arrow). Faint bands of approx. 17 kDa were also observed in *E. coli* protein samples (red arrows), whereby here, several bacterial proteins of higher molecular weight cross-reacted with the anti-H3.5 antibodies.

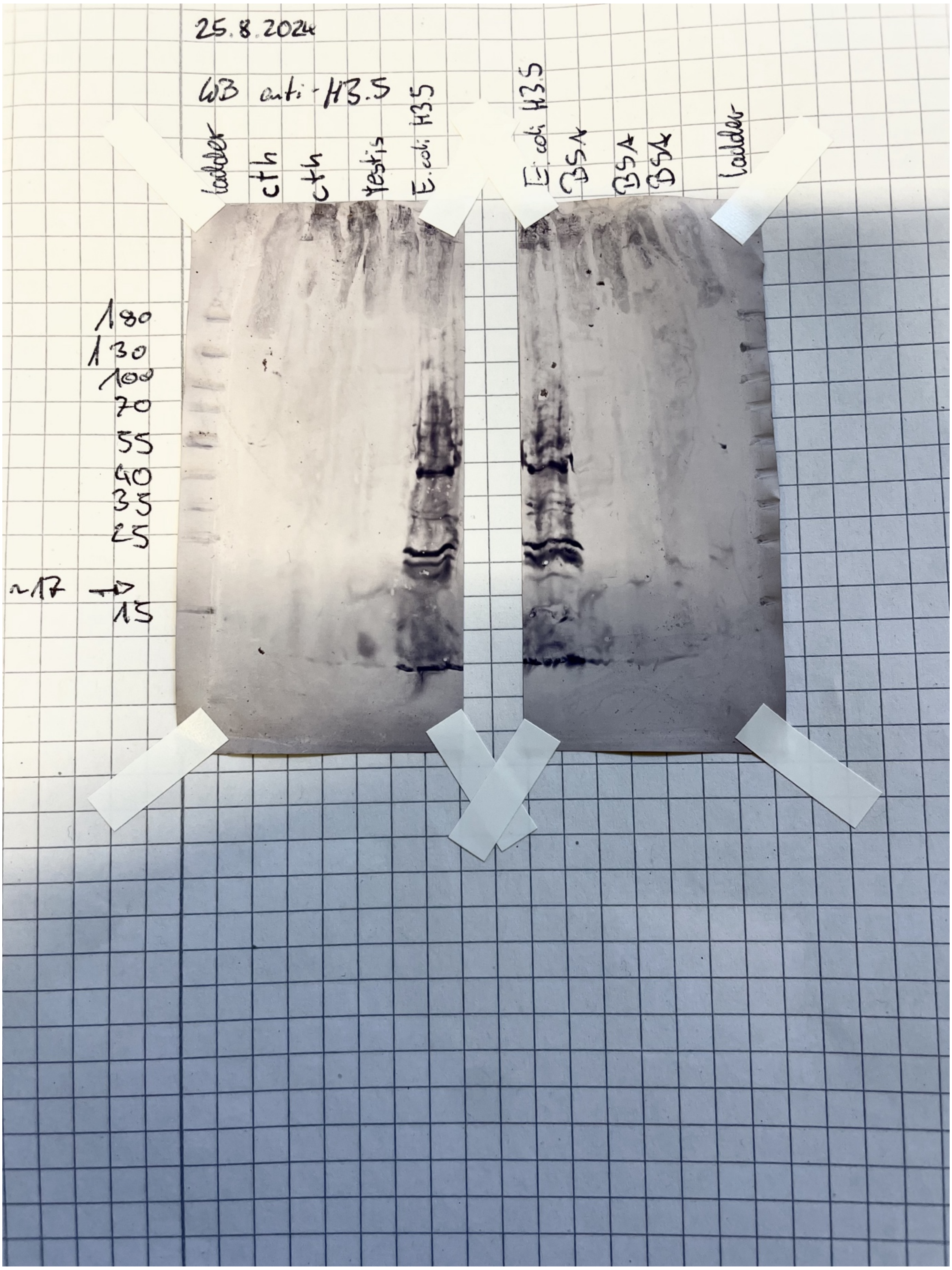

Figure SI1_G. Photograph of the original Western blot membranes used for Figure SI1_F. The staining protocol and steps of image manipulation are described in the caption of Figure SI1_F.
