# Supplementary material for: Histone variant H3.5 in testicular cell differentiation and its interactions with histone chaperones
Source: Sci Rep. 2024 Dec 19;14:30564. doi: 10.1038/s41598-024-83206-9 (PMC11659419; doi:10.1038/s41598-024-83206-9)
Supplement: Supplementary file 4 — Supplementary Material 4 [file 41598_2024_83206_MOESM4_ESM.pdf]

Supplementary Figure S1: Zoomed immunofluorescence microscopy analyses on the localization of H3.5 or pan-H3 in human normal testis sections

Histone Variant H3.5 in Testicular Cell Differentiation and Its Interactions with Histone Chaperones

Patrick Philipp Weil[1], Anton Pembaur[1], Beatrice Wirth[1], Eda Oetjen[1], Hannes Büsscher[1], Klemens Zirngibl[1], Malte Czarnetzki[1], Stella Braun[1], Jann-Frederik Cremers[2], Daniel Gödde[3], Stephan Degener[4], Jan Postberg[1,*]

*corresponding author

[1-4] See main manuscript for details on authors' affiliations.

Figure S1. Zoomed immunofluorescence microscopy analyses on the localization of H3.5 or pan-H3 in human normal testis sections and Hek293 cells. Two areas (**A**. and **B**.) were selected for comparison. The arrows indicate the trajectory of spermatogenesis. Adjacent sections were chosen for image pairs of anti-H3.5 and pan-H3 staining, respectively. **Abbreviations:** Ley, *Leydig cells*; pGc, *prospective germ cells*; sperm, *sperm cells*. Subfigure **C.** shows different zoom levels of a section of the seminiferous tubules. The red channel is a DNA stain. The green channel is a H3.5 staining. Subfigures c1 and c2 show the same section with different combinations of channels for orientation. The white squares mark the area that represents the image section of c3. At these magnification levels, we observed an increasing non-overlap of the fluorescence signals in all axes, x, y and z (chromatic shift) under the experimental conditions at that time, so that H3.5 chromatin patterns are difficult to interpret. Only the partly quite fine-grained structure and the localization in cell nuclei and partly in the cytoplasm of H3.5-positive cells can be confirmed at higher zoom levels. Nevertheless, it is interesting that H3.5 signals presumably show little or no colocalization with the condensed chromosomes in spermatocytes during meiosis. We do not know whether H3.5 has been removed from chromatin at this time or whether it is ready for incorporation (c4).

D. Experiments in which GST-H3.5 was visualized in Hek293 cells are better suited for the investigation of H3.5-associated chromatin patterns. Subfigure D. shows a selection of confocal optical z-sections of 2 adjacent nuclei, in which the extensive but not complete colocalization of GST-H3.5 with the euchromatin marker H3K9ac can be traced. Nucleoli are excluded from staining with both markers. This figure shows a partial reproduction of the experiments on the colocalization of H3.5 with different histone PTMs previously published in [1].

1. Schenk R, Jenke A, Zilbauer M, Wirth S, Postberg J: **H3.5 is a novel hominid-specific histone H3 variant that is specifically expressed in the seminiferous tubules of human testes**. *Chromosoma* 2011, **120**(3):275-285.

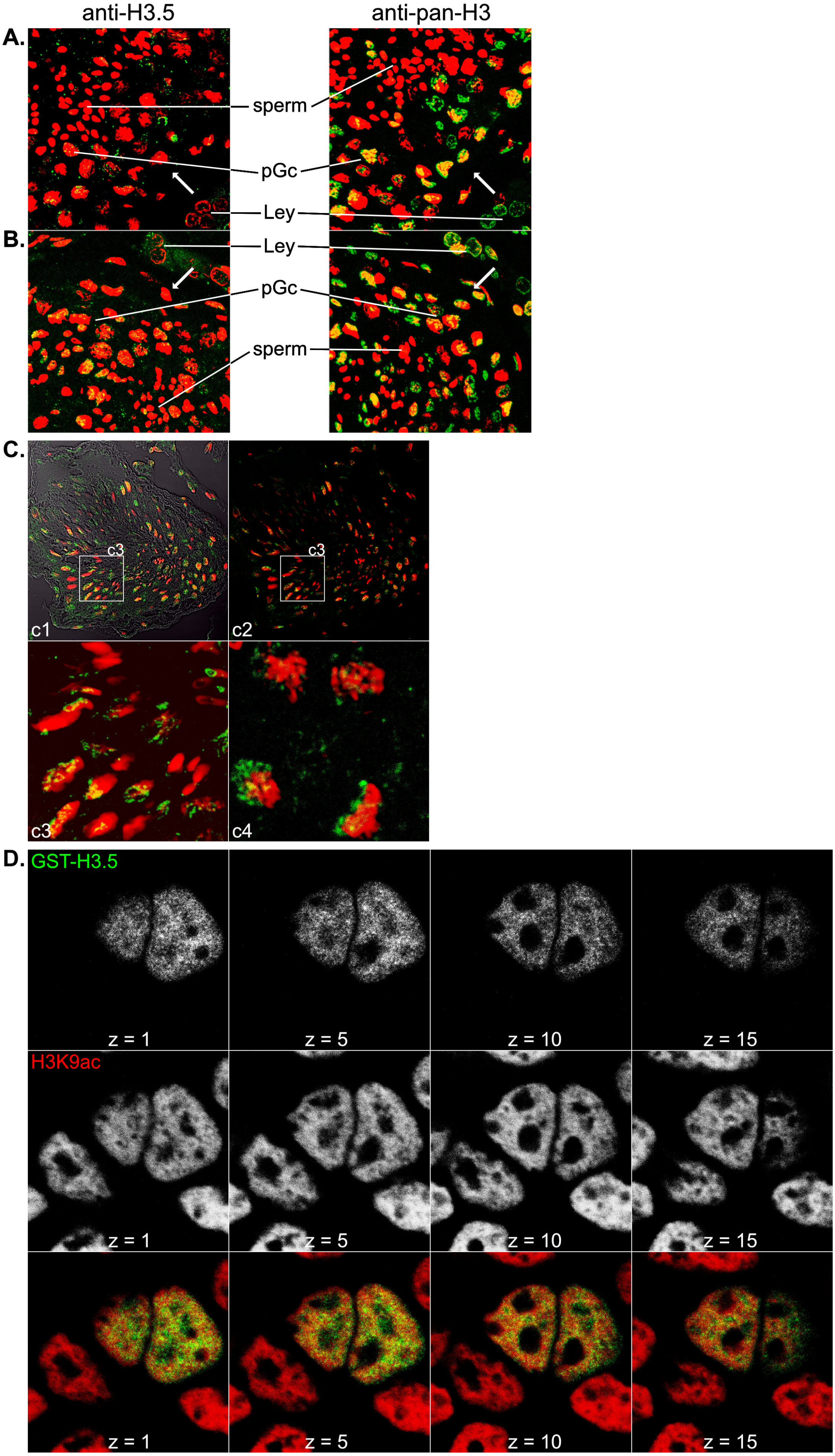

anti-H3.5
anti-pan-H3
A.
sperm
pGc
Ley
B.
Ley
pGc
sperm
C.
c3
c3
c1
c2
c3
c4
D.
GST-H3.5
z = 1
z = 5
z = 10
z = 15
H3K9ac
z = 1
z = 5
z = 10
z = 15
z = 1
z = 5
z = 10
z = 15
